# Case Report: A case of thyroid storm with lower FT3 and FT4 levels accompanied by acute liver failure

**DOI:** 10.3389/fmed.2025.1668176

**Published:** 2025-10-06

**Authors:** Dezhao Li, Feifei Li

**Affiliations:** Department of Infectious Disease, Shandong Provincial Hospital Affiliated to Shandong First Medical University, Jinan, China

**Keywords:** case report, acute liver failure, thyroid storm, corticosteroids, plasma exchange

## Abstract

**Introduction:**

Thyroid storm and acute liver failure (ALF) each independently carry high mortality rates. Previous literature has rarely reported cases of thyroid storm complicated by ALF, particularly in patients with low thyroid hormone levels.

**Patient concerns:**

A 34-year-old woman was admitted to our hospital with fever and jaundice. The patient had been taking methimazole for four months following the diagnosis of hyperthyroidism. Upon admission, Laboratory tests revealed elevated liver enzymes and bilirubin levels. Then, she received liver-protective therapy along with medications to reduce liver enzymes and bilirubin, and antithyroid drugs were temporarily discontinued. Unfortunately, the patient subsequently developed thyroid storm and acute liver failure.

**Diagnosis:**

The patient was diagnosed with thyroid storm associated with acute liver failure.

**Interventions:**

The patient remarkably improved with plasma exchange and corticosteroids treatment and radioactive iodine therapy was performed two months later.

**Outcome:**

The patient was regularly followed up after discharge and remained clinically stable.

**Lessons:**

This case highlights the importance of closely monitoring liver function in patients with hyperthyroidism. In the event of thyroid storm or fulminant liver failure, the use of potentially life-saving interventions such as plasma exchange and corticosteroids should not be delayed.

## Introduction

Thyroid storm is a rare but life-threatening condition with a mortality rate as high as 20 to 30%, with multiple organ failure being the most common cause of death (1). Liver injury associated with thyroid storm can range from mild hepatic enzyme abnormalities to ALF and fulminant hepatitis (2). Approximately 15 to 76% of patients with thyroid storm exhibit mildly elevated liver function, while ALF remains extremely rare in this population (3). The pathogenesis of hepatotoxicity is multifactorial: excessive thyroid hormones exert direct toxic effects on the hepatobiliary system, leading to apoptosis and oxidative stress (4). In addition, a major adverse event associated with antithyroid drug (ATD) therapy is drug-induced liver toxicity (5). Managing hyperthyroid patients with concurrent liver dysfunction remains a therapeutic challenge. In the case presented in this article, ATDs were avoided throughout the treatment process, and therapeutic plasma exchange (TPE) together with corticosteroids was chosen instead. For patients who develop acute liver failure, TPE offers a safe, rapid, and effective treatment option by removing accumulated bilirubin and toxic substances from the body. Notably, a review of this patient’s clinical course suggests that the favorable prognosis was closely associated with the use of corticosteroids.

## Case presentation

**Figure 1:**
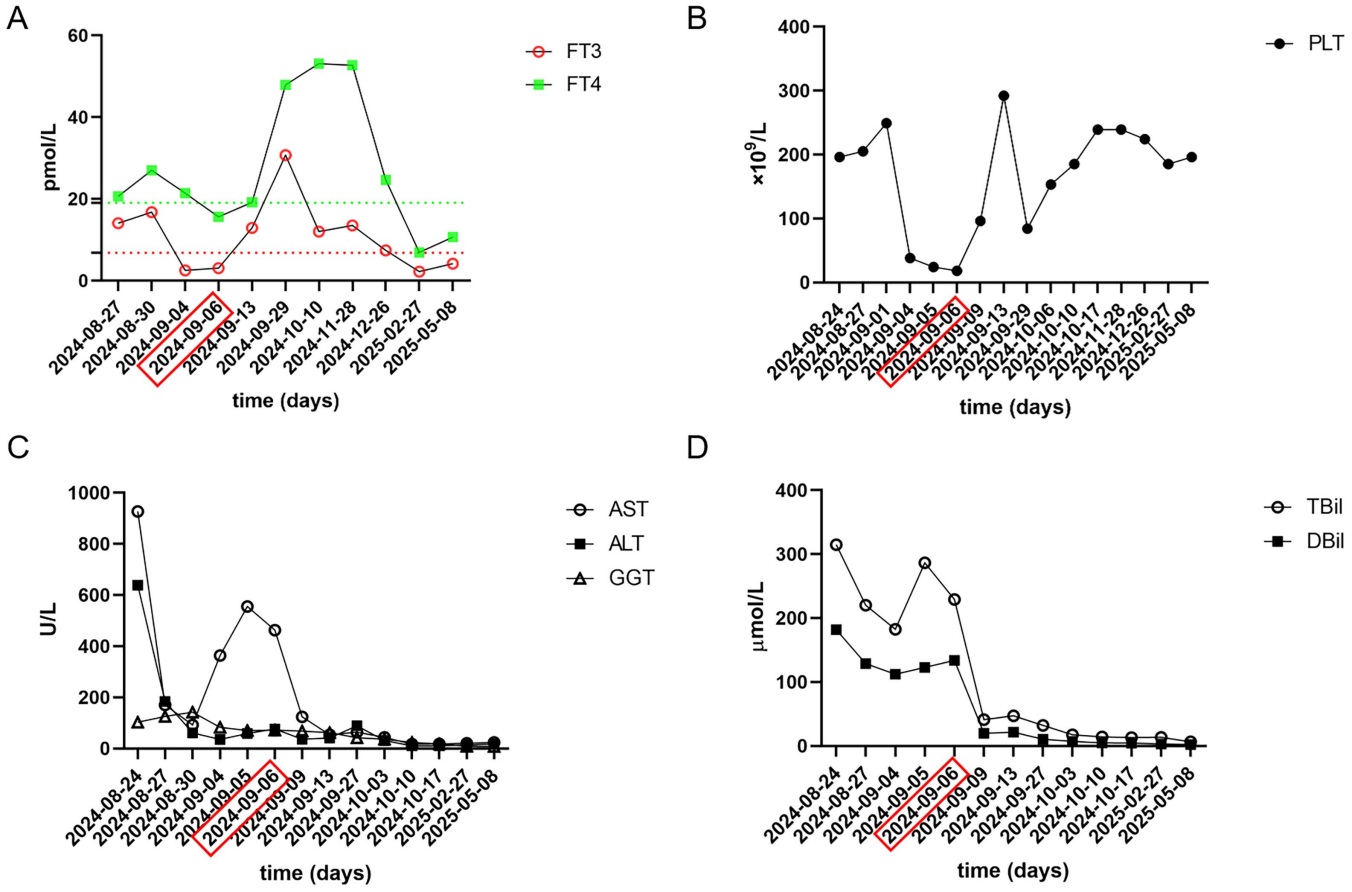
Laboratory data of this patient, obtained during hospitalization. **(A)** FT3, FT4; **(B)** PLT; **(C)** AST, ALT, GGT; **(D)** TBil, DBil. Red dotted line, Upper limit of normal FT3; Green dotted line, Upper limit of normal FT4. PLT, platelet, reference range 125–350 × 109/L; FT3, free triiodothyronine, reference range 2.43–6.01 pmol/L; FT4, free thyroxine, reference range 9.01–19.05 poml/L; AST, aspartate transaminase, reference range 13–35 U/L; ALT, alanine aminotransferase, reference range 7–40 U/L; GGT, Gamma-glutamyltransferase, reference range 7–45 U/L; TBil, total bilirubin, reference range 0–21 μmol/L; DBil, direct bilirubin, reference range 0–4 μmol/L. The date marked by the red rectangle indicates the first therapeutic plasma exchange (TPE), followed by two consecutive days of treatment.

A 34-year-old woman was admitted to our hospital presenting with generalized jaundice and dark-colored urine. Three days prior to admission, she developed a fever with a peak temperature of 39 °C, which responded to antipyretic medications such as ibuprofen. She was evaluated at a local hospital, where laboratory tests revealed abnormal liver function and elevated bilirubin levels. Her past medical history includes a four-month history of hyperthyroidism, for which she has been taking methimazole 20 mg daily and metoprolol succinate extended-release 47.5 mg daily and a diagnosis of Sjögren’s syndrome approximately two months ago, for which she has been taking oral prednisone (15 mg per day). She denied any history of using herbal supplements, alcohol abuse, hepatitis, tuberculosis, or other infectious diseases. On physical examination, the patient had jaundice of the skin and sclera. The thyroid gland was diffusely enlarged. There were no signs of heart failure and the cardiopulmonary auscultation was normal. Laboratory data revealed a thyroid-stimulating hormone of <0.0083 μIU/L (normal range, 0.35–4.94), free triiodothyronine (FT3) of 14.08 pmol/L (normal range, 2.43–6.01), and free thyroxine (FT4) of 20.62 pmol/L (normal range, 9.01–19.05), both exceeding the normal range (Figure 1A). She had serum bilirubin of 314.7 μmol/L (normal range, 0–21) with direct bilirubin of 181.7 μmol/L (normal range, 0–4). Her aspartate transaminase (AST), alanine aminotransferase (ALT) and alkaline phosphatase (ALP) level were 927 IU/L (normal range, 13–35), 639 IU/L (normal range, 7–40) and 162 IU/L (normal range, 35–100), respectively. International normalizing ratio (INR) and prothrombin time were 1.44 (normal range, 0.8–1.2) and 16 s (normal range, 9.4–12.5), respectively. Prothrombin time activity (PTA) was 59% (normal range, 70–140), C-reactive protein (CRP) (normal range, 0–8) and procalcitonin (PCT) (normal range, 0–0.05) levels were elevated (CRP = 15.0 mg/L; PCT = 0.38 ng/mL). All the hepatitis virus markers including IgM serology for hepatitis B virus (HBV), hepatitis A virus (HAV), hepatitis E virus (HEV) were negative. Thyroid ultrasound demonstrated diffuse gland enlargement (right lobe 7.1 × 3.0 × 2.6 cm, left lobe 6.2 × 2.5 × 2.1 cm, isthmus 3.6 × 1.5 × 0.5 cm, pyramidal lobe 2.0 × 0.9 × 0.5 cm), with a thickened capsule and heterogeneous echotexture. Color Doppler showed markedly increased intraparenchymal blood flow. Abdomen MRI showed normal intrahepatic and extrahepatic bile ducts.

**Figure 2:**
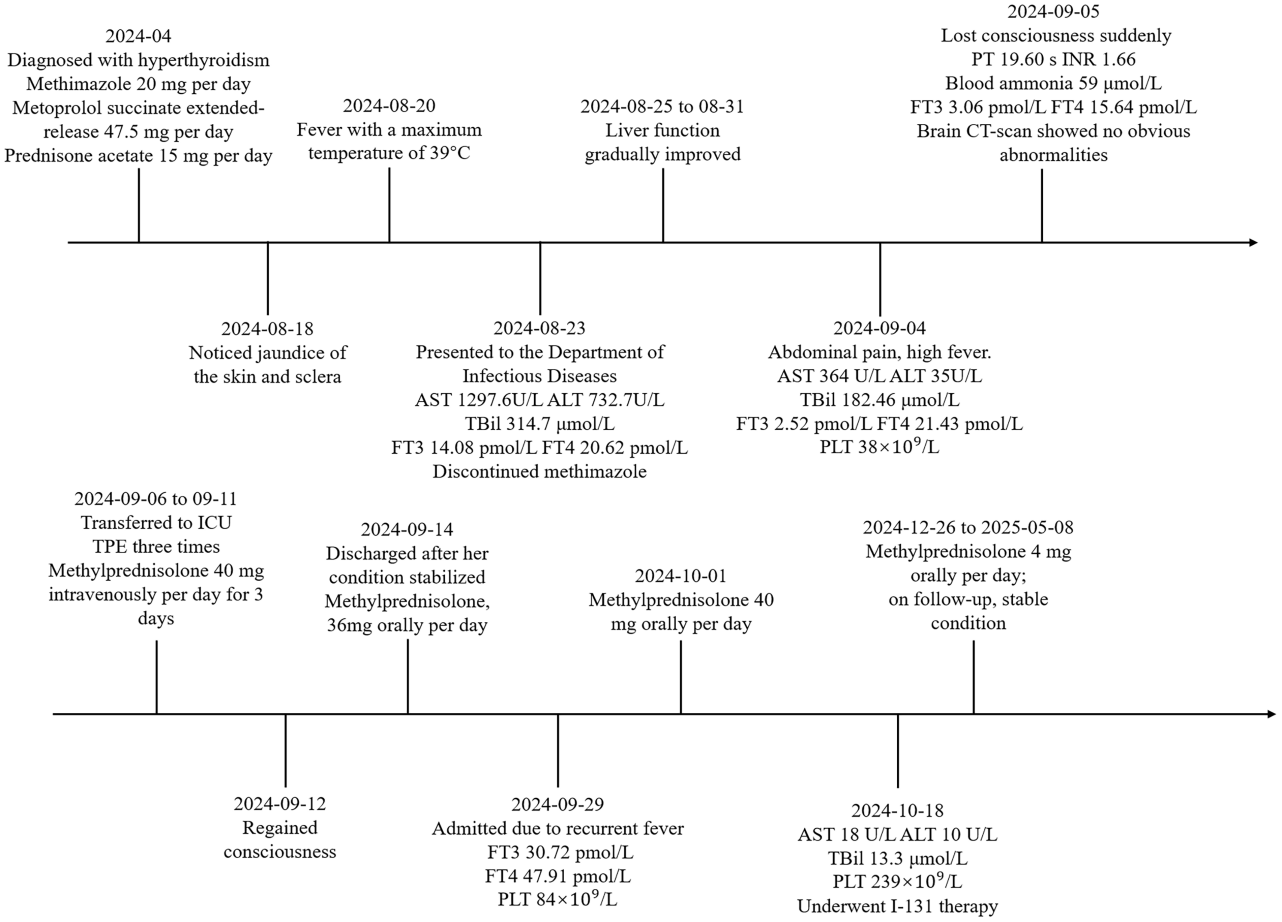
The patient’s treatment process.

Due to severe liver dysfunction, methimazole was discontinued upon the patient’s admission. The treatment regimen included magnesium isoglycyrrhizinate (150 mg, anti-inflammatory), glutathione (1.8 g, detoxification), polyene phosphatidylcholine (20 mL, antioxidant), and ursodeoxycholic acid (250 mg, bile excretion). Oral administration of metoprolol and prednisone acetate was continued. The patient’s liver function gradually improved within one week of admission. However, on the twelfth day of hospitalization, the patient experienced a sudden loss of consciousness. The body temperature was 38.3 °C, the heart rate was 127 bpm, and the blood pressure was 104/72 mmHg. Complete blood test revealed a rapid decline in platelet count (Figure 1B). Liver function were noted to be significantly abnormal with AST of 555 U/L, total bilirubin of 286.34 μmol/L. Her INR, PTA and prothrombin time (PT) were 1.66, 48% and 19.60 s, respectively. Her blood ammonia was 59 μmol/L (normal range, 3–47), consistent with ALF. Her FT3 and FT4 were 3.06 and 15.64 pmol/L. Her thyroid-stimulating hormone was 0.1235 μIU/L. Brain CT-scan showed no obvious abnormalities. ADAMTS13 enzymatic activity and inhibitory antibody tests were negative, thrombotic thrombocytopenic purpura (TTP) was excluded. According to the Burch-Wartofsky Point Scale, the patient meets the criteria for a diagnosis of thyroid storm (Supplementary Table 1). Subsequently, she was transferred to the intensive care unit (ICU), where she received medical management for coma and hyperthermia, including cooling with an ice blanket, enteral nutrition, and prophylactic antibiotics. Following three cycles of TPE and a three-day course of intravenous methylprednisolone sodium succinate at 40 mg per day, the patient’s level of consciousness improved, her bilirubin fell below 50 μmol/L, and slowly spontaneously normalized. She did not have any adverse reaction. After 22 days of hospitalization, the patient was discharged and continued on oral corticosteroid therapy with a gradual and regular tapering of the dose. The initial dose of oral methylprednisolone was 36 mg per day, with a weekly reduction of 4 mg until the dose was reduced to 24 mg. However, fifteen days later, she was readmitted due to a high fever. Her platelet count had again declined to a low level, and her thyroid hormone levels were significantly elevated (Figures 1A,B). The dose of methylprednisolone was increased to 40 mg per day for 2 weeks. Subsequently, the patient’s condition improved, with both liver function and platelet levels returning to normal. Then, she underwent radioactive iodine treatment. We recommended that the patient continue oral methylprednisolone therapy after discharge, starting at 36 mg per day, with a weekly reduction of one tablet until reaching a maintenance dose of 4 mg. During outpatient follow-up, her thyroid hormone and liver enzyme levels remained within the normal range. The trends in her laboratory test results are illustrated in Figure 1. The patient’s treatment process is shown in Figure 2. The use of corticosteroids during the patient’s treatment is shown in Supplementary Figure 1.

## Discussion

Thyroid storm is a life-threatening condition, with an incidence rate of 0.22% and a mortality rate ranging from 8 to 25%, globally. Last estimates from Japan was 10% (6). The diagnosis is primarily clinical, based on the presence of signs and symptoms consistent with severe hyperthyroidism (7). Clinical manifestations include high fever, central nervous system dysfunction, gastrointestinal disturbances such as severe jaundice, tachycardia, congestive heart failure, and atrial fibrillation. Multiple organ failure is the leading cause of death in affected patients (8). The most common trigger of thyroid storm is irregular use or abrupt discontinuation of antithyroid drugs (6, 8, 9). Liver function abnormalities in patients with hyperthyroidism may result either from the direct effects of thyrotoxicosis or from ATD-induced hepatotoxicity, as liver toxicity is a known adverse effect of thioamide medications (10). However, whether to discontinue ATD therapy remains a therapeutic challenge. The American Thyroid Association (ATA) guidelines recommend that ATD treatment should be carefully considered if baseline transaminase levels exceed five times the upper limit of normal (11). Satoh et al. recommend that ATDs should be initiated promptly upon diagnosis of thyroid storm, particularly in patients presenting with impaired consciousness or heart failure (6). When high doses of ATDs are administered, careful monitoring is essential due to the risk of serious side effects such as agranulocytosis and hepatotoxicity (6). It remains unclear whether ATDs should be used in patients with concurrent liver failure. However, high-dose corticosteroids have been shown to inhibit both thyroid hormone synthesis and the peripheral conversion of T4 to T3 (6). A retrospective analysis of this patient’s treatment course suggests that the favorable prognosis was closely linked to corticosteroid therapy. As thyroid storm is a rare endocrine emergency, there are no prospective studies confirming the efficacy of TPE in its management. However, if pharmacological treatment is ineffective, TPE may be considered as an alternative therapy (7). The patient’s clinical deterioration after discharge underscores the role of TPE as a bridge therapy for definitive hyperthyroidism treatment. Patients with thyroid storm complicated by acute liver failure and impaired consciousness are particularly suitable candidates for plasma exchange, as it can help eliminate excessive cytokines associated with systemic inflammatory response syndrome (SIRS).

It is noteworthy that in this patient, the concentrations of FT3 and FT4 were not elevated at the onset of thyroid storm. A study conducted by Akamizu et al. (12) involving 356 patients with thyroid storm found that only one patient had a normal FT4 level with elevated FT3, while six patients had normal FT3 levels with elevated FT4. Notably, no patients had both FT3 and FT4 within the normal range. Even when FT3 and FT4 levels are within the normal range, the possibility of thyroid storm should not be overlooked in patients with hyperthyroidism which was observed in the index case. In cases where only thyroid-stimulating hormone (TSH) levels are suppressed while thyroid hormone levels remain normal, coexisting hyperthyroidism in critically ill patients should be considered (7). Early suspicion, prompt diagnosis, and aggressive treatment are critical to improving survival in patients with thyroid storm. Thyroid storm is an acute condition marked by multiple organ failure and a rapidly progressive clinical course. Therefore, its management requires substantial clinical expertise. In cases complicated by acute liver failure, TPE should be initiated without delay.

This case report has certain limitations. First, follow-up after discharge should have been conducted more closely to better monitor the patient’s condition. Second, a liver biopsy was not performed during the relatively stable phase of the illness, which might have provided additional diagnostic information. Future cases of a similar nature may benefit from more comprehensive diagnostic evaluations and closer follow-up to optimize management strategies.

## Data Availability

The original contributions presented in the study are included in the article/Supplementary material, further inquiries can be directed to the corresponding author.
