# Supplementary material for: Case Report: A case of thyroid storm with lower FT3 and FT4 levels accompanied by acute liver failure
Source: Front Med (Lausanne). 2025 Oct 6;12:1668176. doi: 10.3389/fmed.2025.1668176 (PMC12536029; doi:10.3389/fmed.2025.1668176)
Supplement: Supplementary file 1 [file Data_Sheet_1.docx]

Supplementary Materials

Table 1 The Burch-Wartofsky point scale of our patient^☆^

| **Criteria** | **Points** | **Patient score** |
| --- | --- | --- |
| **Thermoregulatory dysfunction (temperature ℃)** |  | 38.3℃ = 10 |
| 37.2-37.7 | 5 |  |
| 37.8-38.3 | 10 |  |
| 38.4-38.8 | 15 |  |
| 38.9-39.3 | 20 |  |
| 39.4-39.9 | 25 |  |
| ≥40.0 | 30 |  |
| **Cardiovascular** |  | 127 beats per min = 15 |
| **Tachycardia (beats per min)** |  |  |
| 90-109 | 5 |  |
| 110-119 | 10 |  |
| 120-129 | 15 |  |
| 130-139 | 20 |  |
| ≥140 | 25 |  |
| **Atrial fibrillation** |  |  |
| Absent | 0 |  |
| Present | 10 |  |
| **Congestive heart failure** |  |  |
| Absent | 0 |  |
| Mild | 5 |  |
| Moderate | 10 |  |
| Severe | 15 |  |
| **Gastrointestinal-hepatic dysfunction** |  | Jaundice = 20 |
| Absent | 0 |  |
| Moderate (diarrhea, abdominal pain, nausea/vomiting) | 10 |  |
| Severe (jaundice) | 20 |  |
| **Central nervous system disturbance** |  | Seizure = 30 |
| Absent | 0 |  |
| Mild (agitation) | 10 |  |
| Moderate (delirium, psychosis, lethargy) | 20 |  |
| Severe (seizure, come) | 30 |  |
| **Precipitating event Status** |  | Treatment withdrawal = 10 |
| Absent | 0 |  |
| Present | 10 |  |
| **Total score: ≥ 45 thyroid storm** |  | Thyroid storm = 85 |
| Impending storm | 25-44 |  |
| Storm unlikely | ＜25 |  |
| ^☆^Data taken from |  |  |


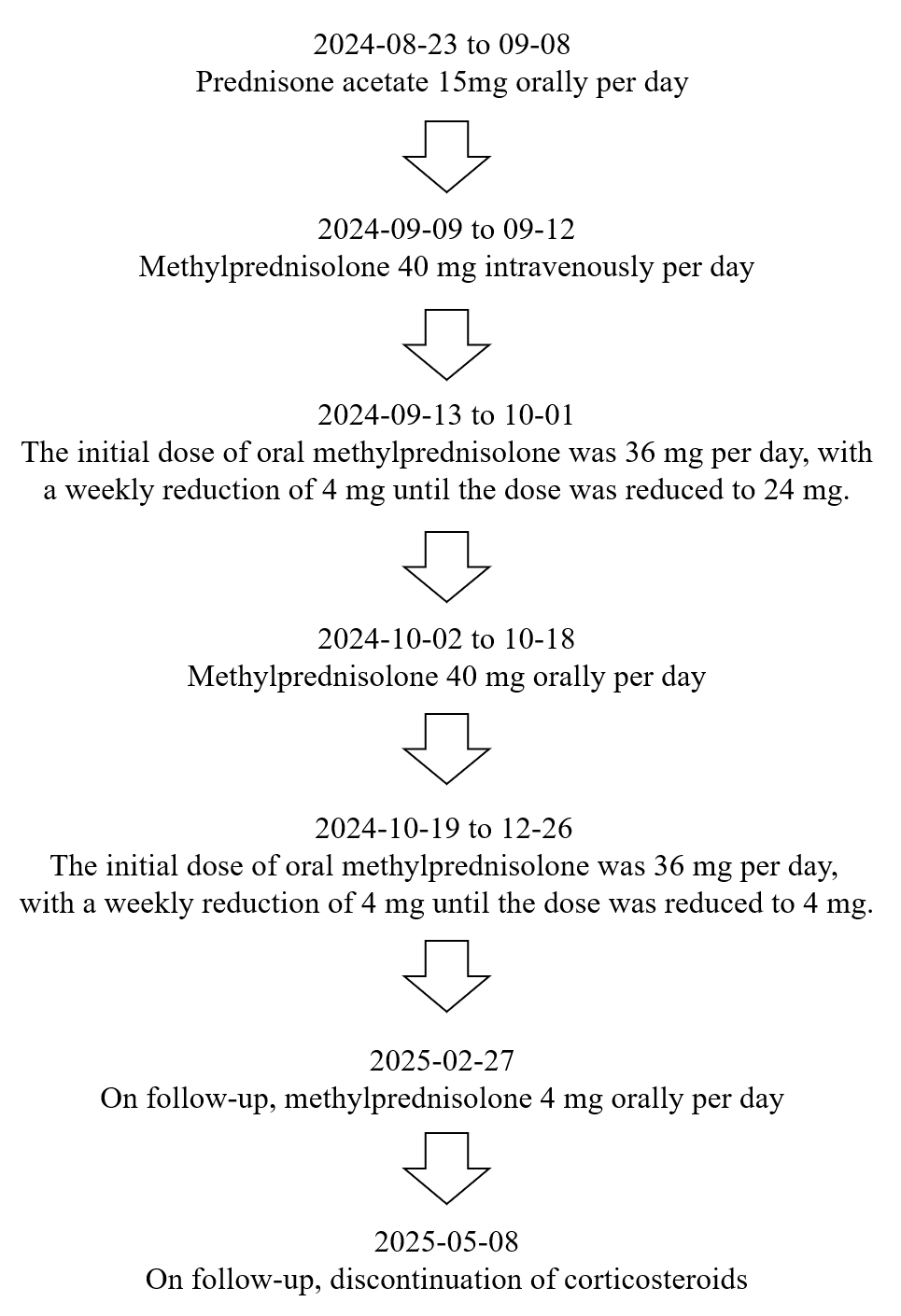


FIGURE 1

The course of corticosteroid administration.
